# First Description of a Large Clinical Series of Fetal Alcohol Spectrum Disorders Children and Adolescents in Reunion Island, France

**DOI:** 10.3390/children11080955

**Published:** 2024-08-07

**Authors:** Laëtitia Sennsfelder, Susie Guilly, Sonia Henkous, Christophe Lebon, Sébastien Leruste, Pauline Beuvain, Fanny Ferroul, Stéphanie Benard, Frédérique Payet, Meissa Nekaa, Maité Bagard, Magaly Lauret, Virginie Hoareau, Aurélie Caillier, Stéphanie Robin, Justine Lanneaux, Léa Etchebarren, Michel Spodenkiewicz, Jean-Luc Alessandri, Godelieve Morel, Bérénice Roy-Doray

**Affiliations:** 1 Laboratoire EPI (Etudes Pharmaco-Immunologiques), UFR Santé, Université de La Réunion, CHU (Centre Hospitalier Universitaire) de La Réunion, 97400 Saint-Denis, France; berenice.doray@chu-reunion.fr; 2 Service de Génétique, CHU (Centre Hospitalier Universitaire) de La Réunion, La Réunion, 97400 Saint-Denis, France; 3 Centre Ressources TSAF (Troubles du Spectre de l’Alcoolisation Fœtale), Fondation Père Favron, CHU (Centre Hospitalier Universitaire) de La Réunion, 97546 Saint-Pierre, France; 4 CIC 1410 (Centre d’Investigation Clinique), CHU (Centre Hospitalier Universitaire) de La Réunion, 97400 Saint-Denis, France; lebon.christophe@hotmail.fr (C.L.); sebastien.leruste@univ-reunion.fr (S.L.); michel.spodenkiewicz@univ-reunion.fr (M.S.); 5 UFR Santé, Université de La Réunion, 97410 Saint-Pierre, France; 6 Centre Diagnostic TSAF (Troubles du Spectre de l’Alcoolisation Fœtale), CHU (Centre Hospitalier Universitaire) de La Réunion, 97400 Saint-Denis, France; 7 Pôle de Santé Mentale, CHU (Centre Hospitalier Universitaire) de La Réunion, 97448 Saint-Pierre, France; 8 Centre de Référence Anomalies du Développement et Syndromes Malformatifs Sud-Ouest Occitanie Réunion, Site Constitutif de La Réunion, 97400 Saint-Denis, France

**Keywords:** fetal alcohol spectrum disorders, clinical description, Reunion Island

## Abstract

Background: Despite several diagnostic guidelines, Fetal Alcohol Spectrum Disorders (FASDs) remain underdiagnosed or misdiagnosed, delaying the care of these patients and support for families. Objective: This study aims to help professionals caring for these children and their families to suspect this diagnosis earlier and to provide the most appropriate follow-up. Methods: A retrospective chart review with monocentric recruitment was performed at the Genetics Unit of the University Hospital of Reunion Island. A total of 147 children and adolescents with FASDs were included. Results: Prenatal alcohol exposure was associated with paternal alcohol consumption in 42.9%, and a high rate of prematurity (33.3%) was observed. Sixty percent of children or adolescents were placed in foster families. Learning difficulties without cognitive deficits were found in 65.8% of cases (50/76). Postural control and fine motor skills disabilities were described, respectively, in 54.7% (35/64) and 72.5% (50/69) of cases. A systematic genetic assessment was carried out, identifying in these FASD patients an associated Copy Number Variation (CNVs) in 22.6% of cases. Conclusion: Children with FASDs combine significant vulnerabilities, associating exposure to alcohol during the preconception and/or the prenatal period, prematurity, complex familial and sociocultural living conditions, and a genetic anomaly in almost a quarter of cases.

## 1. Introduction

Fetal Alcohol Spectrum Disorders (FASDs) are considered a major public health condition and are the leading cause of avoidable neurocognitive disorders and social maladjustment, with a worldwide prevalence of 7.7 per 1000 births. Fetal Alcohol Syndrome (FAS), Partial Fetal Alcohol Syndrome (pFAS), Alcohol-Related Neurodevelopmental Disorders (ARND) and Alcohol-Related Birth Defects (ARBD) represent the continuum of adverse effects of prenatal alcohol exposure on the fetus. FAS is the most visible and complete form, including growth failure, characteristic facial features (short palpebral fissures, smooth philtrum and thin upper lip), neurobehavioural impairment and structural brain alterations. pFAS is associated with neurobehavioural impairment, characteristic facial features and, if prenatal alcohol exposure (PAE) is unknown, growth failure or brain malformation. The absence of characteristic facial features characterizes ARND, and only neurobehavioural impairment is observed with confirmed PAE. ARBD is mentioned when PAE is documented with specific major malformations described in animal models [1,2,3]. The highest prevalence was found in the WHO European region (19.8 per 1000) and lowest in the WHO Eastern Mediterranean region (0.1 per 1000) [4]. Concerning the individual countries, the highest FASD prevalence was found in South Africa (111.1 per 1000), Croatia (53.3 per 1000), Ireland (47.5 per 1000), Italy (45.0 per 1000) and Belarus (36.6 per 1000). More importantly, 76 countries had a prevalence of FASDs > 1%, exceeding the USA prevalence of neurodevelopmental conditions such as Down syndrome (trisomy 21), Edwards syndrome (trisomy 18), spina bifida and anencephaly. Among special populations, the prevalence of FASDs was 5.2 to 67.7 times higher in children with medicosocial care, 30.3 times higher in the inmate population, and 23.7 times higher in a low socioeconomic status population, compared with the global prevalence among children and adolescents in the general population [5,6,7,8]. In France, the estimated global prevalence was 10.4 per 1000 persons [6]. In 2018, the French Public Health Agency determined that the overall Fetal Alcohol Syndrome (FAS) frequency was 0.48 per 1000 births in France between 2006 and 2013, but Reunion Island showed the highest prevalence with 1.22 per 1000 births—more than two and a half times the national rate.

With a prevalence of 10 per 1000 persons, more than 10% of women in the United States declared at least some alcohol consumption during pregnancy [9]. According to the 2017 French health barometer, even occasional consumption of alcohol during pregnancy would concern approximately one in ten pregnant women [10]. Worldwide, alcohol consumption by pregnant women has been estimated at 10%. The highest prevalence was found in the WHO European region, which is consistent with the FASD prevalence [11]. International guidelines advise against drinking any amount or type of alcohol during this period. This is all the more important, as 47% and 39% of pregnancies are unplanned in developing and developed countries, respectively [12]. Several factors have been identified as associated with alcohol use by mothers during pregnancy: they included higher gravidity and parity, delayed pregnancy recognition, inadequate prenatal care or reluctance of health professionals to address alcohol use, a history of FASDs in previous children, alcohol use disorder and other substance use, mental health disorders, a history of physical or sexual abuse, social isolation, intimate partner violence, alcohol and/or drug use during pregnancy by the mother’s partner, or others family members, and low socioeconomic level [13,14,15,16,17]. 

Prenatal alcohol exposure (PAE) not only affects the developing fetus but also the placenta. Abnormal placentation is observed, increasing the risk of miscarriage or fetal death in utero, bleeding and prematurity, gestational arterial hypertension, intrauterine growth restriction (IUGR) and premature rupture of membranes (PRM) [18,19].

The physical, mental and social consequences for individuals with FASDs can negatively impact their social environment, daily life, school, relationships and work [20]. However, despite specific guidelines, the diagnosis of FASDs remains complex due to several factors. First, the risk and the severity of FASDs depend not only on the time, dose, frequency and duration of maternal alcohol consumption but also on the age, gravidity, parity, nutritional status, and maternal and fetal genomes [21]. Additionally, FASDs are often confused with other genetic syndromes such as velocardiofacial syndrome (VCFS), also known as DiGeorge syndrome, Williams syndrome or Cornelia de Lange syndrome [22,23,24]. The non-visible forms such as ARND are often confused with attention deficit hyperactivity disorder (ADHD), whereas executive dysfunction is greater in FASD children compared to children with ADHD [25]. Moreover, the diagnosis of children under 3 years is complex since reliable neuropsychological testing cannot be performed, and there is a lack of alcohol consumption biomarkers with a specificity and sensibility of 100% [26,27]. Despite research into miRNAs and DNA methylation as more sensitive and specific biomarkers of FASDs, results are preliminary and require further investigation [28,29]. FASDs will continue to be underdiagnosed or misdiagnosed and based on clinical evidence until new biomarkers with better sensitivity and specificity are found.

Consequently, this study aimed to describe a large series of FASD children and adolescents from Reunion Island. Our main objective is to help professionals caring for these children and their families to help professionals make this diagnosis early and implement the most appropriate follow-up, considering that a FASD diagnosis before the age of 6 offers a better prognosis [30].

## 2. Materials and Methods

### 2.1. Study Population

**Figure 1:**
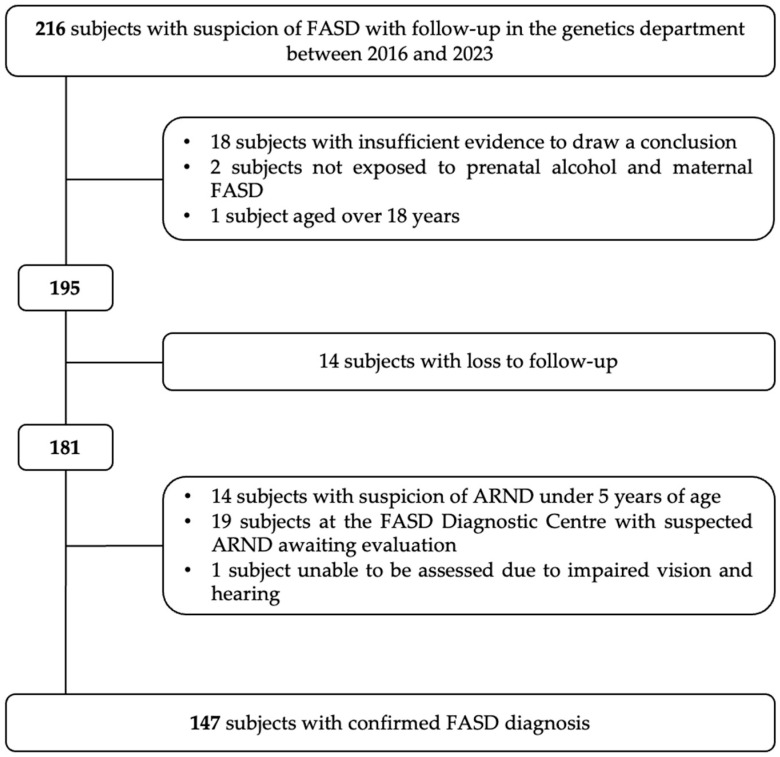
Flow chart of subject inclusion between 2016 and 2023.

A retrospective chart review with monocentric recruitment was performed at the Genetics Unit of the University Hospital of Reunion Island. Medical records of all patients with a confirmed diagnosis of FASDs between 2016 and 2023 and aged between 0 and 18 years were included. A total of 147 children and adolescents with FASDs from Reunion Island were included (Figure 1). A request for compliance with the reference method (MR004) was submitted to the “Commission Nationale de l’Informatique et des Libertés”, registered under number 2024-A00029-38. All study participants were sent an information sheet to inform them about using their medical data.

### 2.2. Study Design and Setting

The diagnosis of FAS and pFAS was established by a physician specializing in FASDs or after a multidisciplinary assessment at the FASDs diagnostic center. ARND was only diagnosed at the diagnostic center after a multidisciplinary assessment from the age of 5 years. The 4-digit diagnostic code (based on growth failure, FAS facial phenotype, central nervous system (CNS) structural/functional abnormalities and PAE) was used to classify FASD children into FAS, pFAS and ARND. Genetic analyses, including chromosomal microarray analysis by comparative genomic hybridization array (CGH array) or single nucleotide polymorphisms array (SNP array) and molecular analysis for X-fragile syndrome, were also systematically proposed. In addition, a malformative assessment was performed, including brain MRI, cardiac and abdomino–pelvic ultrasounds, and sensory examination. The neuropsychological assessment was performed using the Wechsler Intelligence Scale for Children for children from 5 years of age and the Wechsler Adult Intelligence Scale for adolescents from 16 years of age. To determine the cognitive profile (IQ), the cognitive competence index was used, but it may have proven to be uninterpretable and not representative of the patient’s real abilities due to heterogeneous profiles: in this case, the index of general aptitude was calculated.

The receptive and productive aspects of language were also assessed. Cognitive functions, such as verbal comprehension, fluid reasoning, processing speed and working memory, were assessed, as were executive functions (planning, inhibition, cognitive flexibility). The psychomotor evaluation included the establishment of psychomotor level and the search for motor impairments such as postural problems, balance and fine motor skills. Finally, behavioral abnormalities such as attention/concentration difficulties, impulsivity, emotional dysregulation, aggressive behavior and motor hyperactivity were analyzed.

Clinical, biological and radiological data were entered into an anonymized spreadsheet and analyzed using simple frequency analysis with R studio software 2022.02.3 Build 492.

## 3. Results

The main results of the following sections are summarized in Table 1.

### 3.1. Description of the Population

This series of children and adolescents with FASDs was divided into 19% (*n* = 28/147) of FASD children under 5 years and 81% (n= 119/147) of FASD children or adolescents between 5 and 18 years. Among these 28 FASD children under 5 years, 75% (*n* = 21/28) were diagnosed with FAS, whereas 25% (*n* = 7/28) of them were diagnosed with partial FAS by a specialized practitioner before the evaluation at the diagnostic center. The other 119 FASD children were classified as 37% (*n* = 44/119) of FAS, 36.1% (*n* = 43/119) of partial FAS, and 26.9% (*n* = 32/119) of ARND. A total of 12.9% of the mothers of the children in our study had FASDs themselves.

### 3.2. Alcohol Consumption

Maternal alcohol consumption was established in 99.3% of the cases (*n* = 146/147), either after discussions with the mother or after information obtained from the socio-educational actors (without maternal confirmation). In one FAS patient, only paternal alcohol consumption in the preconception period was known. The precise prenatal period of consumption was known for 72 of 147 cases. Among these 72 cases, the consumption concerned the first trimester only in 31.9% (*n* = 23/72), the first and the second trimester in 16.7% (*n* = 12/72), and the whole pregnancy in 51.4% (*n* = 37/72). When the pregnancy was known, only 36.1% (*n* = 22/61) of the mothers stopped their alcohol consumption. Different types of alcoholic beverages were consumed during the pregnancy, such as beer (31.5%; *n* = 17/54), wine (9.3%, *n* = 5/54), distilled beverages (9.3%; *n* = 5/54), and multiple consumption (50.0%, *n* = 27/54). The maternal consumption frequency was classified as daily (59.5%; *n* = 50/84), festive/occasional (10,7%, *n* = 9/84), once a week (1.2%, *n* = 1/84), 2–3 times a week (1.2%, *n* = 1/84), >2–3 times a week (4.8%, *n* = 4/84), and on the weekend (22.7%, *n* = 19/84).

This prenatal alcohol exposure (PAE) was associated with tobacco in 38.1% (56/147), cannabis in 10.9% (16/147), and medications in 4.1% (6/147) of the cases.

In addition to maternal alcohol consumption, paternal alcohol consumption was declared in 42.9% of the cases (*n* = 63/147).

### 3.3. Course of Pregnancy and Birth

Most of the mothers discovered their pregnancy during the first or the second trimester, 46.5% (*n* = 20/43) and 44.2% (*n* = 19/43), respectively. A total of 9.3% (*n* = 4/43) of them discovered their pregnancy in the third trimester. The mean maternal age at delivery was 26.4 years. A total of 6.7% (*n* = 6/89) of the women were under 18 years. The rate of prematurity was 33.3% (*n* = 45/135), with 19.2% (*n* = 26/135) of late preterm, 5.2% (*n* = 7/135) of moderate preterm, 2.2% (*n* = 3/135) of very preterm, and 6.7% (*n* = 9/135) of extremely preterm birth.

### 3.4. Socio and Family Environment

A total of 24.5% of FASD children or adolescents (*n* = 36/147) lived with their mothers, 4.8% (*n* = 7/147) with their fathers, and only 9.5% (*n* = 14/147) with their two biological parents. A total of 59.2% (*n* = 87/147) were placed; 0.7% (*n* = 1/147) was adopted. Children were mainly placed in foster families (74.7%; *n* = 65/87) before the mean age of 3.4, with multiple placements in 33.3% (*n* = 29/87) of cases.

### 3.5. Medical and Genetic Data

Apart from the characteristic facial dysmorphia comprising at least two out of the three criteria and identified in 115 of 147 patients, a camptodactyly, most often reducible, associated with hypotonia of the distal muscles of the extensors of the fingers, was identified in 21 out of 147 patients (8 patients with FAS, 7 patients with partial FAS and 6 patients with ARND). Camptodactyly is a rare, congenital disorder characterized by painless, often bilateral, non-traumatic and congenital permanent flexion of the proximal interphalangeal joint. Two brothers suffering from a severe form of FAS presented with true distal arthrogryposis requiring the wearing of splints.

A microcephaly was present at birth in 39.6% (38/96) of cases. In addition, brain structural malformations were observed in 27.7% (*n* = 23/83) of cases. These 23 malformations concerned the corpus callosum for 10 cases (agenesia (1/10), hypoplasia (8/10), dysplasia (1/10)); the cerebellar vermis (hypoplasia) for 6 cases; the hippocampus (hypoplasia) for 2 cases; and the insula, the amygdala (ptosis), the pituitary gland and thalami in one case each; one case of holoprosencephaly and one case of neurinoma were also observed. A congenital heart defect (ventricular septal defect) was observed in 7.8% of cases (*n* = 8/103). Abdomino–pelvic ultrasound was abnormal in 4.5% of cases (*n* = 4/88), visualizing one case of splenomegalia and three cases of kidney abnormalities (two renal hyperplasia, one ectopia). An abnormal ENT was observed in 15.8% of cases (*n* = 12/76).

Concerning genetic analyses, a copy number variation (CNV) was identified in 23.5% (*n* = 27/115) of the patients.

### 3.6. Neuropsychological and Psychomotor Assessments

Seventy-six FASD children or adolescents benefited from a multidisciplinary assessment at the FASD Diagnostic Centre, including neuropsychological and psychomotor tests. The average age of children with FASDs at the FASD diagnostic center was 9.5 years. A mental deficiency was described in 34.2% of cases (*n* = 26/76). In 47.4% of cases (*n* = 36/76), an intellectual disability was not observed. These data were not available for 14 of the 76 subjects: for 5 patients, the IQ was not calculated because of the absence of several data; for 7 patients, the results were too heterogeneous to calculate the IQ; for 1 patient, the IQ was lacking without explanation. The average intellectual level of patients without associated CNV was 63.8 among the FAS patients, 75.5 among the pFAS patients and 80.2 among the ARND patients. Learning difficulties without cognitive deficiency were identified in 65.8% of the cases (*n* = 50/76), especially in verbal comprehension; 63.2% (*n* = 48/76) in fluid reasoning; 73.7% (*n* = 56/76) in working memory; and 56.6% (*n* = 43/76) in processing speed. In addition, executive function abnormalities were present, such as planification in 46.1% (*n* = 35/76) of cases, mental flexibility in 65.8% (*n* = 50/76) of cases and lack of inhibition in 25% (*n* = 19/76) of cases. Receptive and productive oral language difficulties were described in 61.8% (*n* = 47/76) and 68.4% (*n* = 52/76), respectively. Concerning the psychomotor evaluation, a psychomotor delay was observed in 75% (*n* = 57/76). Motor impairments like postural control and fine motor skills were described in 46.1% (*n* = 35/76) and 65.8% (*n* = 50/76), respectively. Behavioral abnormalities were dominated by attention/concentration disorder (72.4%; *n* = 55/76), impulsivity (48.7%; *n* = 37/76), emotional dysregulation (69.7%; *n* = 53/76), aggressive behavior (47.4%; *n* = 36/76) and motor hyperactivity (56.6%; *n* = 43/76).

## 4. Discussion

This study demonstrated the consequences of teratogenic and neurotoxic effects due to prenatal alcohol consumption. This series of children and adolescents with FASDs was divided into 44.2% (*n* = 65/147) of subjects with FAS, 34% (*n* = 50/147) of subjects with pFAS, and 21.8% (*n* = 32/147) of subjects with ARND.

Interestingly, this study highlighted a lesser-known, non-pathognomonic sign of FASDs, namely camptodactyly of the fingers. This sign is probably underdiagnosed and has little research, although according to our series and our experience, it has an obvious diagnostic interest. This rate was consistent with the literature, which estimates the prevalence of camptodactyly in FASDs between 12 and 54% [31]. This camptodactyly, linked to a distal muscular deficit of the second phalanges, will lead to a lack of strength in the ends of the fingers, a lack of precision, a less effective thumb grip, difficulties with fine motor skills and graphic disorders. Specific psychomotor or occupational therapy support is necessary and effective.

Our results confirmed the existence of a recognizable cognitive–behavioural profile of these children. Cognitive difficulties were frequently reported in our study, involving verbal comprehension; fluid reasoning; working memory; processing speed; and executive functions such as planning, cognitive flexibility and inhibition. These results were consistent with the literature [32,33,34]. This very specific neuropsychological profile constitutes a valuable aid in suspecting the diagnosis, particularly in the absence of characteristic facial dysmorphism [32]. Behavioral abnormalities, attention/concentration difficulties, impulsivity, emotional dysregulation, aggressive behavior and motor hyperactivity were present in children or adolescents with FASDs in this study.

This descriptive study also highlighted that FASD children or adolescents had cumulative vulnerabilities. The first associated factor of vulnerability concerns the effects of paternal alcohol consumption and parental consumption of other products, particularly teratogenic or neurotoxic products, in the future child. In our study, prenatal alcohol exposure due to maternal consumption was frequently associated with paternal alcohol consumption (42.9% of cases). While much of the focus over the past year has been on the effects of maternal consumption, there is evidence of the harmful effects of paternal consumption before conception, especially by epigenetic alterations of the sperm, concerning anomalies of DNA methylation or of the expression of several sncRNAs including tRNA-derived small RNA (tDR), mitochondrial small RNA and miRNAs [28,35,36,37,38]. Epigenetic states are not reset in developing germ cells, and purely epigenetic states can be maintained in the germ line for one or more generations, surviving periods of epigenetic resetting [39].

Beyond these biological aspects, psychosocial aspects within the couple cannot be neglected: it was demonstrated that paternal drinking was highly correlated with maternal drinking during pregnancy. In the study of Bakhireva et al., 51.2% of pregnant women continued to drink during pregnancy if their partners were heavy episodic or frequent drinkers, compared with 17.1% of women whose partners were not categorized as frequent or heavy episodic drinkers (*p* < 0.0001) [40].

Other exposures during pregnancy, as possible factors of vulnerability, were examined, such as tobacco and cannabis. In our study, tobacco was present in 38.1% of the pregnancies (56/147), cannabis in 10.9% (16/147) and medications in 4.1% (6/147).

Both alcohol and cannabis are known as neuroinhibitory drugs, with the particularity that cannabis synergistically enhances the effects of alcohol. Alcohol and cannabis act on inhibitory interneurons in the adult hippocampal formation and affect inhibitory processes in the dentate gyrus during adult neurogenesis. These two molecules act on the same signaling pathways during early brain development, including the sonic hedgehog pathway, which is important for closing the neural tube and establishing neural identity in the ventral part of the spinal cord and hindbrain [41,42].

Consequently, health professionals should pay attention to paternal alcohol consumption but also to the consumption of other toxic products.

Prematurity is a second associated vulnerability, representing 33.3% (45/135) of births in our study. This rate was twice as high as the rate of prematurity in Reunion (12.5%) and four times higher than in mainland France (7.5%) [43]. Alcohol consumption during pregnancy, especially in the first few weeks, is associated with a high risk of vascular-placental damage, which is responsible for an increased risk of early miscarriage, premature birth, low birth weight and stillbirth. Hypervascularization and increased diameter of umbilical arteries in the placenta, a reduction in placental vascular density and luminal area of placental blood vessels, and a reduction in the number and luminal area of small villi were observed. Immunostaining for placental growth factor (PLGF) and vascular endothelial growth factor receptors 1 and 2 (VEGFR1/2), which are involved in blood vessel formation and growth, was also reduced [12,19,39,42]. In addition, PAE reduces PLGF levels, thereby altering the fetal brain vasculature. Placental suppression of PLGF altered VEGF-R1 expression in the brain, mimicking alcohol-induced vascular defects in the cortex [41]. As a result, these pregnancies should be considered by health professionals as high-risk pregnancies.

Maternal age constitutes an interesting question: the mean maternal age at birth was 26.4 years in our series, which is lower than the average maternal age in the Reunionese population (29.7 years) [44]. Women under the age of 18 represented 6.7% (6/89) of the cases, which is twice their rate in the total population of Reunion (2.7%) [45].

In our series of FASD children or adolescents, 61.4% were placed before the age of 3.4, with multiple placements in 33.3% of cases. This repartition is clearly different in mainland France, with 76% of adopted children, 17% of placement and 7% of children living with their biological parents [46]. In our opinion, based on our past professional experience in mainland France but also discussions with the “Vivre avec le SAF” Association, these differences reflect the fact that most of the children diagnosed in mainland France are adopted children, particularly from Eastern Europe, and that the identification of FASDs, in the absence of an adoptive context, remains very low.

Because of this high placement rate, health professionals do not necessarily have the opportunity to meet mothers and obtain details about their alcohol consumption. This may explain the high rate of unavailability of data on maternal alcohol consumption (duration, type, frequency) and the course of the pregnancy (gestational age at pregnancy diagnosis, living conditions, intrafamily violence). Placement is often associated with misdiagnosis. For example, the study by Chasnoff et al. showed that 80.1% of children in a sample of foster and adopted children with FASDs aged 4 to 18 years had never been diagnosed, and 6.4% had been misdiagnosed [7]. In addition, FASDs may be 10–15 times more common in foster and adopted children than in the general population; these children are particularly at risk because many are removed from their homes due to parental substance use [47,48,49]. In addition, multiple placements may increase the risk of absent or inappropriate medical, social, and educational care, and this population shows a higher incidence of learning disabilities, disrupted school experiences, inappropriate sexual behavior, trouble with the law, and increased risk of substance abuse later in life in these children or adolescents with FASDs; the risk of misdiagnosis is increased, whereas early intervention before the age of 6 years can significantly improve their outcome [30,49,50].

The last important associated vulnerability is the presence of associated genetic anomalies in FASD children. We previously demonstrated, on a smaller series of patients, a rate of 20.8% of CNVs [51]. After continuing this previous study, we found an updated rate of 23.5% CNVs. This rate was higher than other rates found in the literature [52,53]. It reinforces the plea for a multidisciplinary approach to developmental disorders, exploring both environmental factors, such as avoidable teratogens, and intrinsic vulnerabilities, especially genetic determinants; it also suggests a causal link between alcohol consumption and the occurrence of chromosomal micro-rearrangements. In addition to a diagnosis of FASDs, these children may have a CNV that influences the phenotype, potentially accentuating or reducing certain signs. For example, in our series of FASD children or adolescents, if the average intelligence quotient of patients with ARND was 80.2, it decreased to 71 in ARND patients with CNV. Interestingly, the facial dysmorphia of patients suffering from FAS but also carrying a CNV can be modified or even attenuated, complicating the establishment of the diagnosis. Some of these CNVs can also be found in chromosomal regions that contain genes regulating the epigenetic machinery, which is already disrupted by prenatal alcohol exposure. These could also influence the phenotype and, therefore, the severity of these disorders. A total of 12.9% of the mothers of the children in our study had a known diagnosis of FASDs. In the majority of cases, there is a well-known transgenerational alcohol consumption; nevertheless, in our experience, some mothers carrying FASDs, in the absence of alcohol consumption during pregnancy, have children with growth retardation but also learning disorders (difficulties with memorization, attention) raising the important question of the transmission of epigenetic abnormalities to their children.

To conclude, this study is the first to examine such a large series of FASD patients on Reunion Island. In addition to the well-known teratogenic and neurobehavioral consequences of preconception and prenatal alcohol exposition, our children with FASDs presented associated vulnerabilities such as prematurity, difficult emotional and sociocultural living conditions, and genetic abnormalities. The presence of these vulnerabilities in a child should be a red flag to healthcare professionals and raise the possibility of FASDs.

Therefore, all mothers and fathers should be questioned about alcohol use and also cannabis and tobacco use during the mother’s pregnancy. The simultaneous presence of these following signs should prompt healthcare professionals to assess the following for FASDs: prematurity, foster care, microcephaly, facial dysmorphia (small palpebral fissures, long and smooth philtrum, thin upper lip) and camptodactyly; cognitive difficulties highly dominated by disorders of verbal comprehension, fluid reasoning, working memory, processing speed, and executive functions; and behavioral abnormalities including especially, attention/concentration difficulties, impulsivity, emotional dysregulation, aggressive behavior and motor hyperactivity.

This study also reinforces the importance of early multidisciplinary care for alcohol-exposed pregnancy and FASD patients to improve their medical prognosis, limit associated vulnerabilities and secondary disabilities, and optimize their quality of life. The benefit also concerns the rest of the family, sometimes including brothers and sisters who may also be affected, and systematically the parents through information and appropriate care. With an average age at diagnosis of 9.4 years, children with FASDs are still diagnosed too late, especially for ARND. One of the perspectives is to find epigenetic biomarkers to contribute to the early identification of affected children.

## Figures and Tables

**Table 1:** Main results of this study.

Period	Item	Sub-Items	Results
Prenatal	Alcohol consumption	Period of pregnancy	1st trimester: 31.9% (*n* = 23/72) 1st and 2nd trimester: 16.7% (*n* = 12/72) Whole pregnancy: 51.4% (*n* = 37/72)
		Types of alcoholic beverages	Beer: 31.5% (*n* = 17/54) Wine: 9.3% (*n* = 5/54) Distilled beverages: 9.3% (*n* = 5/54) Multiple consumption: 50.0% (*n* = 27/54)
		Maternal consumption frequency	Daily: 59.5% (*n* = 50/84) Festive/occasional: 10.7% (*n* = 9/84) Once a week: 1.2% (*n* = 1/84) 2–3 times a week: 1.2% (*n* = 1/84) >2–3 times a week: 4.8% (*n* = 4/84) Weekend only: 22.7% (*n* = 19/84)
		Other toxic products associated with maternal consumption	Tobacco: 38.1% (56/147) Cannabis: 10.9% (16/147) Drugs: 4.1% (6/147)
	Course of pregnancy and birth	Pregnancy discovery	1st trimester: 46.5% (*n* = 20/43) 2nd trimester: 44.2% (*n* = 19/43) 3rd trimester: 9.3% (*n* = 4/43)
		Prematurity	Late preterm: 19.2% (*n* = 26/135) Moderate preterm: 5.2% (*n* = 7/135) Very preterm: 2.2% (*n* = 3/135) Extremely preterm: 6.7% (*n* = 9/135)
Postnatal	Family and social environment		Living with their mother: 24.5% (*n* = 36/147) Living with their father: 4.8% (*n* = 7/147) Living with their two biological parents: 9.5% (*n* = 14/147) Foster children: 59.2% (*n* = 87/147) Adopted children: 0.7% (*n* = 1/147)
	Medical and genetic data		Microcephaly: 39.6% (*n* = 38/96) Brain structural malformation: 27.7% (*n* = 23/83) Congenital heart defect: 7.8% (*n* = 8/103) Camptodactyly: 14.3% (*n* = 21/147) Abnormal abdomino–pelvic ultrasound: 4.5% (*n* = 4/88) Abnormal ENT: 15.8% (*n* = 12/76) Copy number variation rate: 23.5% (*n* = 27/115)
	Neuropsychological and psychomotor assessments	Cognitive difficulties	Mental deficiency: 34.2% (*n* = 26/76) Verbal comprehension difficulties: 65.8% (*n* = 50/76) Fluid reasoning difficulties: 63.2% (*n* = 48/76) Working memory difficulties: 73.7% (*n* = 56/76) Processing speed difficulties: 56.6% (*n* = 43/76)
		Executive functions	Planification difficulties: 46.1% (*n* = 35/76) Mental flexibility difficulties: 65.8% (*n* = 50/76) Lack of inhibition: 25% (*n* = 19/76)
		Oral language difficulties	Receptive: 61.8% (*n* = 47/76) Productive: 68.4% (*n* = 52/76)
		Motor impairment	Psychomotor delay: 75% (*n* = 57/76) Postural control difficulties: 46.1% (*n* = 35/76) Fine motor skills difficulties: 65.8% (*n* = 50/76)
		Behavioral abnormalities	Attention/concentration disorder: 72.4% (*n* = 55/76) Impulsivity: 48.7% (*n* = 37/76) Emotional dysregulation: 69.7% (*n* = 53/76) Aggressive behavior: 47.4%, (*n* = 36/76) Motor hyperactivity: 56.6% (*n* = 43/76)

## Data Availability

Individual data are unavailable due to privacy restrictions.
